# Up-regulated expression of substance P in CD8^+^ T cells and NK1R on monocytes of atopic dermatitis

**DOI:** 10.1186/s12967-017-1196-6

**Published:** 2017-05-01

**Authors:** Zenan Zhang, Wenjiao Zheng, Hua Xie, Ruonan Chai, Junling Wang, Huiyun Zhang, Shaoheng He

**Affiliations:** 1 Allergy and Clinical Immunology Research Centre, The First Affiliated Hospital of Jinzhou Medical University, No. 2, Section 5, Renmin Street, Guta District, Jinzhou, 121001 Liaoning People’s Republic of China; 2 Department of Laboratory Medicine, Zibo Mining Group Co., LTD. Central Hospital, Zibo, 255120 Shandong China; 3 The PLA Center of Respiratory and Allergic Disease Diagnosing Management, General Hospital of Shenyang Military Area Command, Shenyang, 110840 Liaoning China

**Keywords:** Allergen, NK1R, T cell, Substance P

## Abstract

**Background:**

Large numbers of CD8^+^ T cells were observed in atopic dermatitis (AD) skin, and monocytes from AD patients showed increased prostaglandin E2 production. However, little is known about the expression of substance P (SP) and its receptor NK1R in blood leukocytes of patients with AD.

**Objective:**

To explore the expression of SP and NK1R in leukocytes of AD and the influence of allergens on SP and NK1R expression.

**Methods:**

The expression levels of SP and NK1R in patients with AD were examined by flow cytometry, ELISA and a mouse AD model.

**Results:**

The plasma SP level was 4.9-fold higher in patients with AD than in HC subjects. Both the percentage of SP expression in the population and mean fluorescence intensity (MFI) of SP expression were elevated in CD8^+^ T cells in the blood of AD patients. However, both the CD14^+^NK1R^+^ population and MFI of NK1R expression on CD14^+^ cells were enhanced in the blood of AD patients. Allergens ASWE, HDME and PPE failed to up-regulate SP expression in CD8^+^ T cells. However, allergens ASWE and HDME both enhanced NK1R expression on CD14^+^ blood leukocytes regardless of AD or HC subjects. OVA-sensitized AD mice showed an elevated proportion and MFI of SP-expressing CD8^+^ T cells in the blood, which agrees with the SP expression situation in human AD blood. Injection of SP into mouse skin did not up-regulate NK1R expression on monocytes.

**Conclusions:**

An elevated plasma SP level, up-regulated expression of SP and NK1R indicate that the SP/NK1R complex is important in the development of AD. Therefore, SP and NK1R antagonist or blocker agents may help to treat patients with AD.

*Trial registration* Registration number: ChiCTR-BOC-16010279; Registration date: Dec., 28, 2016; retrospectively registered

## Background

AD is a chronic inflammatory skin disease that affects up to 25% of children and 10% of adults [1]. The pathogenesis of AD is multi-factorial and complex. T helper (Th) 2-driven inflammation is thought to play a significant role in the pathogenesis of AD [1]. Group 2 innate lymphoid cells and Th17 dominance may also be involved in AD [2]. However, little is known about the involvement of CD8^+^SP^+^ T cells and SP-expressing monocytes in AD.

CD8^+^ T cells are a significant and previously unappreciated source of inflammatory cytokines, including IL-13, IFN-γ, and IL-22 in AD, and a large number of CD8^+^ T cells was observed in AD skin [3]. It is also observed that adult AD has increased frequencies of IL-22-producing CD4 and CD8 T cells within the skin-homing population [4] and that significantly elevated autoreactive CD8^+^ T cells in AD are effector memory type cells [5]. IL-13 is a key Th2 cytokine involved in atopic disease, while IFN-γ and IL-22 play pivotal roles in host defence against microbes and in the development of chronic inflammatory diseases [6]. Thus, CD8^+^ T cells likely contribute to the development of AD. However, the relationship between SP and CD8^+^ T cells in AD remains unknown.

Monocytes may also be involved in the development of AD as they can act as antigen-presenting cells in AD [7] and produce increased amounts of inflammatory mediator prostaglandin E2 in AD patients [8]. Monocytes can also produce inflammatory mediator SP [9]. Since peripheral blood mononuclear cells (PBMCs) from AD patients proliferate at a high rate [10] and human monocyte/macrophage cell line THP-1 expresses functional SP receptor NK1R [11], it is likely that SP and NK1R expressing monocytes are enhanced in patients with AD.

The SP/NK-1 receptor complex has been recognized as an integral part of the microenvironment of inflammation and is involved in the molecular basis of many human pathologies, [12] including chronic spontaneous urticaria [13] and intestinal fibrogenesis after chronic colitis [14]. However, little is known about the involvement of SP and NK1R in the development of AD. Since SP is a potent proinflammatory mediator [15] and activation of NK1R can amplify a proinflammatory response [16], it is likely that SP and NK1R are involved in AD.

The relationship between allergens and SP in AD has not been previously reported. However, reports have found that mite allergen patching induces allergic dermatitis [17], nasal allergen methacholine provocation rapidly induces bronchial hyperresponsiveness via pulmonary up-regulation of SP and activation of NK1 receptors [18], and allergic inflammation induces substance P synthesis in the neurons of guinea pig lungs [19]. All these findings implicate that allergens may affect SP expression in AD.

Therefore, the aim of the present study is to investigate the expression of SP and NK1R in blood leukocyte CD4^+^, CD8^+^, CD16^+^, CD19^+^, CD123^+^HLA-DR^−^ and CD14^+^ cells of AD patients in parallel and the influence of allergens on SP and NK1R expression in these blood leukocytes.

## Methods

### Reagents

The following compounds were purchased from Biolegend (San Diego, CA, USA): Red Blood Cell Lysis Buffer, APC/Cy7-conjugated mouse anti-human CD4, PE/Cy7-conjugated mouse anti-human CD8, PE/Cy7-conjugated mouse anti-human CD14, PerCP-conjugated mouse anti-human CD16, APC/Cy7-conjugated mouse anti-human CD19, PE-conjugated mouse anti-human CD123, PerCP-conjugated mouse anti-human HLA-DR, PE/Cy7-conjugated rat anti-mouse CD8α, BV421-conjugated rat anti-mouse CD11b, PerCP/Cy5.5-conjugated rat anti-mouse CD49b, FITC-conjugated Armenian hamster anti-mouse FcεRIα, APC/Cy7-conjugated rat anti-mouse Ly6C, APC-conjugated streptavidin, Zombie Aqua™ Fixable Viability Kit, human Fc receptor blocking solution, and anti-mouse CD16/32. FITC-conjugated mouse anti-human SP antibody was purchased from Lifespan (Rochester, NY, USA); and its isotype antibody FITC-conjugated rabbit IgG was obtained from eBioscience (San Diego, CA, USA). APC-conjugated mouse anti-human NK1R, Human SP ELISA kit (sensitivity: 8.04 pg/ml), and its isotype antibody, APC-conjugated mouse IgG3 were supplied by R&D Systems (Minneapolis, MN, USA). PE-conjugated anti-mouse NK1R antibody was bought from Novus (Saint Charles, Missouri, USA). Rat anti-mouse SP antibody was purchased from Fitzgerald Industries International (Bodmin, UK) and was biotinylated by Top-Peptide Bio-Technology Co. Ltd. (Shanghai, China). A Cytofix/Cytoperm™ Kit was obtained from BD Pharmingen (San Jose, CA, USA). SP, brefeldin A and ovalbumin (OVA, Grade V) were purchased from Sigma-Aldrich (St Louis, MO, USA). Foetal bovine serum (FBS, HyClone) and RPMI 1640 were from Gibco BRL (Grand Island, NY, USA). *Artemisia sieversiana* wild allergen extract, house dust mite allergen extract, and *Platanus* pollen allergen extract were purchased from Macro Union Pharmaceutical Co. Ltd. (Beijing, China). Allergens for skin prick tests were supplied by ALK-Abelló, Inc. (Denmark). Most of the general chemicals, such as salts and buffer components, were of analytical grade.

### Patients and samples

A total of 26 AD and 16 healthy control (HC) subjects were recruited in the study. Their general characteristics are summarized in Table 1. The diagnostic criteria of AD were confirmed by the criteria suggested by Kang and Tian [20]. Food allergy and drug allergy were diagnosed based on the criteria suggested by the National Institute of Allergy and Infectious Diseases (NIAID) [21] and the National Clinical Guideline Centre [22]. Informed consent from each volunteer, according to the Declaration of Helsinki and in agreement with the ethical committee of the First Affiliated Hospital of Jinzhou Medical University, was obtained.

**Table 1:** Characteristics of adult subjects

Population	Case	Age (years)	Female/male	History (years)	Onset age (years)
HC	16	26 (25–30)	10/6	0	0
AD	26	46 (18–78)	16/10	4.5 (0.08–17)	45 (16–65.6)
Pollen (+)	6	32.5 (20–49)	4/2	4.9 (0.08–10.83)	27.58 (10.16–43)
Mite (+)	4	36 (24–52)	3/1	2.5 (0.5–3)	33 (23.5–50)
Food (+)	6	47 (18–78)	3/3	2 (0.5–5.16)	47.5 (17.5–77.5)
Small molecule (+)	9	44 (19–69)	5/4	4 (0.5–17)	43 (12–68.5)
Cockroach (+)	1	22	1/0	0.08	21.92

Median values (range) are shown. Specific allergens were examined by skin prick test

*HC* healthy control, *AD* atopic dermatitis

Blood from each patient and from HC subjects was collected in the outpatient clinic. From each individual, 10 ml of peripheral blood was collected into an EDTA-containing tube before centrifugation at 450×*g* for 10 min. The cells were used for flow cytometric analysis, and plasma was collected and frozen at −80 °C for further use.

### Animals

BALB/c mice (4–6 weeks, 18–22 g) were obtained from Vital River Laboratory Animal Technology Co. Ltd. (Beijing, China), Certificate No 11400700118760. The animals were bred and reared under strict ethical conditions according to international recommendations. They were housed in the Animal Experimental Center of the First Affiliated Hospital of Jinzhou Medical University in a specific pathogen-free environment with free access to standard rodent chow and water, at a constant temperature of 23–28 °C and relative humidity of 60–75%. The animal experiment procedures were approved by the Animal Care Committee at Jinzhou Medical University.

### Flow cytometric analysis of SP and NK1R expression in human blood leukocytes

To detect expression of SP and NK1R in human blood leukocytes, blood cells were resuspended in RPMI 1640 medium supplemented with 3% (v/v) heat-inactivated FBS and 100 units/ml penicillin/streptomycin. Cells were then stimulated with or without *A. sieversiana* wild allergen extract (ASWE), house dust mite allergen extract (HDME), or *Platanus* pollen allergen extract (PPE) (all at concentrations of 0.1 and 1.0 μg/ml) for 1 h at 37 °C, respectively, after which 2 μg/ml brefeldin A was added to each tube at the same point. Cells were washed and resuspended in PBS, and specific staining (Zombie Aqua™ Fixable Viability KIT) to exclude the dead cell population [23] and human Fc receptor blocking solution were included according to the manufacturer’s instructions. Cells were divided into two tubes: Tube 1, PE/Cy7-conjugated anti-human CD8, PE-conjugated anti-human CD123, and PerCP-conjugated anti-human HLA-DR antibodies were added; Tube 2, PE/Cy7-conjugated anti-human CD14, PerCP-conjugated anti-human CD16 and APC/Cy7-conjugated anti-human CD19 antibodies were added for 15 min at room temperature. After lysing with Red Blood Cell Lysing buffer, leukocytes were fixed and permeabilized by using a Cytofix/Cytoperm™ Fixation/Permeabilization Kit according to the manufacturer’s instructions. Following washing, FITC-conjugated anti-human SP and APC-conjugated anti-human NK1R antibodies were added to both tubes and incubated at 4 °C for 30 min.

Finally, cells were washed and resuspended in fluorescence-activated cell sorting (FACS)-Flow solution and analysed with a FACS Verse flow cytometer (BD Biosciences, San Jose, CA). A total of 10,000 events in the first positive gate were analysed for each sample. The data were analysed with FlowJo software version 7.0 (Treestar, Ashland, USA). Dead cells and doublets were excluded from analysis by live/dead cell dyes.

### Flow cytometric analysis of SP and NK1R expression in mouse blood leukocytes

To detect expression of SP and NK1R in basophils, neutrophils, monocytes and CD8^+^ T cells of mouse blood, a specific staining (Zombie Aqua™ Fixable Viability Kit) to exclude the dead cell population [23] and anti-mouse CD16/32 were included according to the manufacturer’s instructions. Antibodies against cell surface markers, including PE/Cy7-conjugated anti-mouse CD8α, BV421-conjugated anti-mouse CD11b, PerCP/Cy5.5-conjugated anti-mouse CD49b, FITC-conjugated anti-mouse FcεRIα and APC/Cy7-conjugated anti-mouse Ly6C, were added for 15 min at room temperature. After lysing red blood cells, leukocytes were fixed and permeabilized by using the Cytofix/Cytoperm™ Fixation/Permeabilization Kit as described above. The cell pellets were resuspended, and PE-conjugated anti-mouse NK1R and biotin-conjugated SP were added at 4 °C for 30 min, after which APC-streptavidin was added for 30 min. The FACS analysis was performed as described above.

### Mouse sensitization and challenge

The mouse AD model was mainly adopted from a previous study by Spergel [24] with modest modification. Briefly, female mice were anesthetized with ether, then shaved with a razor blade before stimulation with a dermaroller (soaked in 1.0 mg/ml OVA solution before contacting skin) five times per day, which mimicked an AD skin lesion inflicted by scratching. Each mouse received the treatment every other day for 3 weeks. For control experiments, healthy mice only received NS instead of OVA solution. One week after the last stimulation, animals were euthanized, and their blood was collected into an EDTA-containing tube before centrifugation at 450×*g* for 10 min. The cells were used for flow cytometric analysis, and plasma was collected and frozen at −80 °C for further use. For certain mice, 50 μl of SP (at a concentration of 10 ng/ml) were injected subcutaneously around the skin lesion area for 3 h before animals were euthanized.

### Statistical analysis

Statistical analyses were performed by using SPSS software (Version 17.0, IBM Corporation). The data for expression of SP and NK1R on leukocytes and plasma levels of SP are presented as a scatter plot, in which a horizontal line indicates the median value. The data for expression of SP and NK1R on leukocytes upon allergen challenge are displayed as a boxplot, which indicates the median, interquartile range, and the largest and smallest values for the number of experiments indicated. For all analyses, *P* < 0.05 was considered significant.

## Results

### Levels of SP in patients with AD

**Fig. 1:**
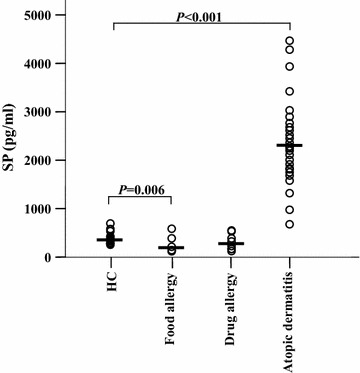
Scatter plots of levels of substance P (SP) in the plasma of patients with atopic dermatitis (AD), food allergy, drug allergy and healthy control (HC) subjects. Each *symbol* represents the value from one subject. The median value is indicated by a *horizontal line*. *P* < 0.05 was considered statistically significant

Elevated plasma levels of SP in AD patients have been previously reported [25, 26]. To confirm the involvement of SP in AD, we examined the plasma levels of SP in patients with AD and food and drug allergies. Using a commercial ELISA kit, we observed that the SP plasma level was 4.9-fold higher (2349 vs. 392 pg/ml) in patients with AD than in HC subjects (Fig. 1). In contrast, SP levels were not elevated in the plasma of patients with drug allergy (306 pg/ml) and were markedly reduced in patients with food allergy (153 pg/ml) in comparison with the plasma SP level of HC subjects (Fig. 1).

### Expression of SP in peripheral blood leukocytes of patients with atopic dermatitis

**Fig. 2:**
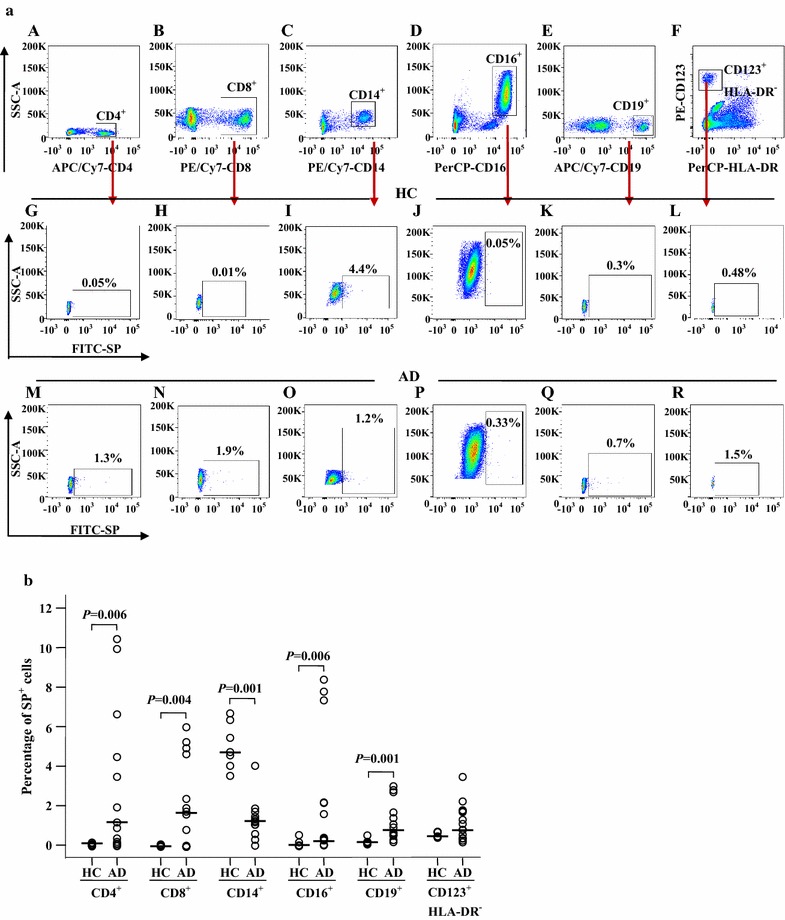
Flow cytometry analysis of expression of substance P (SP) in the peripheral blood leukocytes of patients with atopic dermatitis (AD) and healthy control (HC) subjects. **a** Representative graphs of the percentage of SP^+^ cells out of the corresponding leukocyte population. (*A*), (*B*), (*C*), (*D*), (*E*) and (*F*) show the gating strategies of different leukocyte populations indicated. (*G*), (*H*), (*I*), (*J*), (*K*) and (*L*) represent SP^+^ populations of CD4^+^ (helper T cells), CD8^+^ (cytotoxic T cells), CD14^+^ (monocytes), CD16^+^ (neutrophils), CD19^+^ cells (B cells), and CD123^+^HLA-DR^−^ (basophils) in HC subjects, respectively. (*M*), (*N*), (*O*), (*P*), (*Q*) and (*R*) represent SP^+^ populations of CD4^+^, CD8^+^, CD14^+^, CD16^+^, CD19^+^, and CD123^+^HLA-DR^−^ cells in patients with AD, respectively. **b** The median values of the percentage of SP^+^ cells from AD and HC subjects. Each *symbol* represents the value from one subject. The median value is indicated by a *horizontal line*. *P* < 0.05 was taken as statistically significant

**Fig. 3:**
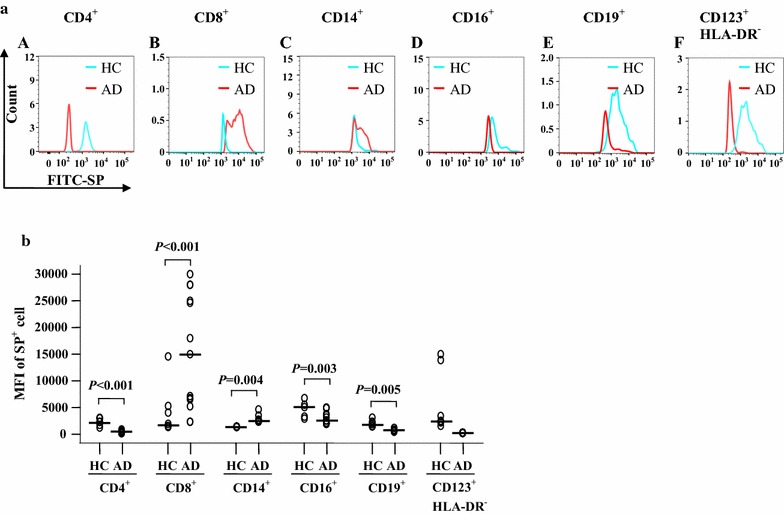
Flow cytometry analysis of expression of substance P (SP) in the peripheral blood leukocytes of patients with atopic dermatitis (AD) and healthy control (HC) subjects. **a** Representative graphs of the changes in mean fluorescence intensity (MFI) of SP in (*A*) CD4^+^, (*B*) CD8^+^, (*C*) CD14^+^, (*D*) CD16^+^, (*E*) CD19^+^, and (*F*) CD123^+^HLA-DR^−^ cells, respectively, in AD and HC subjects. **b** The median values of MFI of SP^+^ cells from AD and HC subjects. Each *symbol* represents the value from one subject. The median value is indicated by a *horizontal line*. *P* < 0.05 was considered statistically significant

To identify a potential source of elevated plasma SP, we investigated the expression of SP in peripheral blood leukocytes of patients with AD. The results showed that percentages of SP-expressing CD4^+^, CD8^+^, CD16^+^, and CD19^+^ cells, but not CD123^+^HLA-DR^−^ and CD14^+^ cells, were increased in the blood of AD patients compared with HC blood (Fig. 2a, b). However, MFI of SP expression in CD8^+^ and CD14^+^ cells, but not CD4^+^, CD16^+^, CD19^+^ and CD123^+^HLA-DR^−^ cells, were enhanced in the blood of AD patients compared with HC blood (Fig. 3a, b). Since both the percentage of the SP-expressing population and the MFI of SP expression were elevated in CD8^+^ T cells, the increased plasma level of SP in patients with AD was likely caused by CD8^+^ T cells.

### Expression of NK1R in peripheral blood leukocytes of patients with atopic dermatitis

**Fig. 4:**
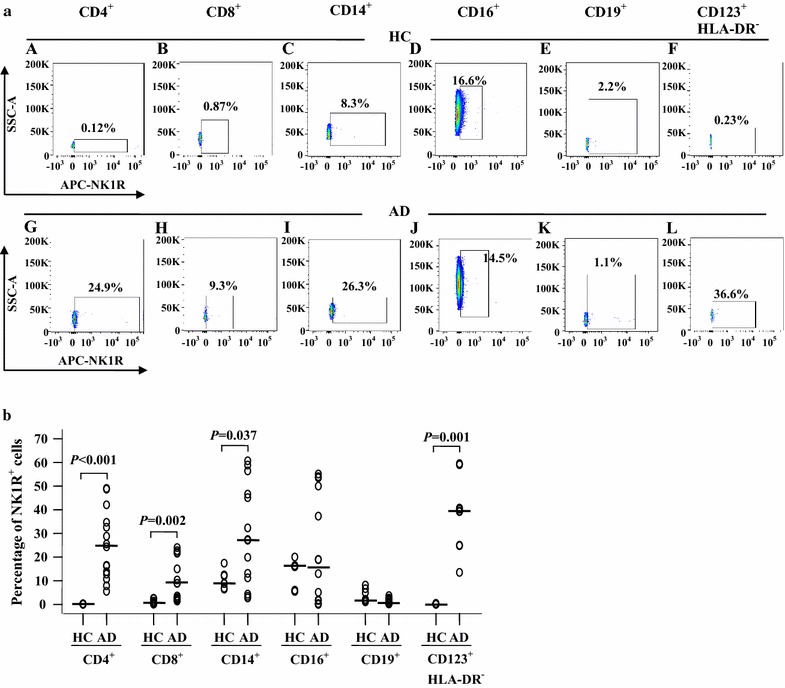
Flow cytometry analysis of expression of NK1R in peripheral blood leukocytes of patients with AD and healthy control (HC) subjects. **a** Representative graphs of the percentage of NK1R^+^ cells out of the corresponding leukocyte population. (*A*), (*B*), (*C*), (*D*), (*E*) and (*F*) represent NK1R^+^ populations of CD4^+^ (helper T cells), CD8^+^ (cytotoxic T cells), CD14^+^ (monocytes), CD16^+^ (neutrophils), CD19^+^ (B cells), and CD123^+^HLA-DR^−^ cells (basophils) in HC subjects, respectively. (*G*), (*H*), (*I*), (*J*), (*K*) and (*L*) represent NK1R^+^ populations of CD4^+^, CD8^+^, CD14^+^, CD16^+^, CD19^+^, and CD123^+^HLA-DR^−^ cells in patients with AD, respectively. **b** The median values of the percentage of NK1R^+^ cells from AD and HC subjects. Each *symbol* represents the value from one subject. The median value is indicated by a *horizontal line*. *P* < 0.05 was considered statistically significant

**Fig. 5:**
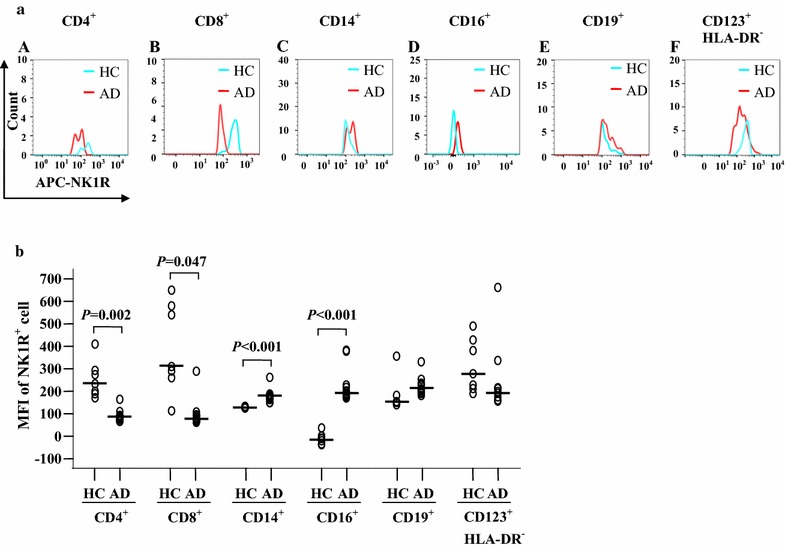
Flow cytometry analysis of expression of NK1R in peripheral blood leukocytes of patients with AD and healthy control (HC) subjects. **a** Representative graphs of the changes in mean fluorescence intensity (MFI) of NK1R in (*A*) CD4^+^, (*B*) CD8^+^, (*C*) CD14^+^, (*D*) CD16^+^, (*E*) CD19^+^, and (*F*) CD123^+^HLA-DR^−^ cells, respectively, in AD and HC subjects. **b** The median values of MFI of NK1R^+^ cells from AD and HC subjects. Each *symbol* represents the value from one subject. The median value is indicated by a *horizontal line*. *P* < 0.05 was considered statistically significant

Since NK1R is a receptor of SP found in the nervous system and in peripheral tissues [27] and its expression level affects SP-provoked cell responses and SP-involved diseases, we investigated the expression of NK1R on the blood leukocytes of patients with AD in the present study. The results showed that the percentages of NK1R^+^ cells in CD4^+^, CD8^+^, CD14^+^ and CD123^+^HLA-DR^−^, but not CD16^+^ and CD19^+^ cells, were elevated in the blood of AD patients in comparison with HC blood (Fig. 4a, b). In terms of the MFI of NK1R expression, the blood of AD patients showed increases in CD14^+^ and CD16^+^ cells but a clear decrease in CD4^+^ and CD8^+^ cells compared to HC subjects (Fig. 5a, b). Since both the percentage of the NK1R-expressing population and the MFI of NK1R expression were elevated in CD14^+^ cells, the increased plasma level of SP may act on blood CD14^+^ cells in patients with AD.

### Induction of expression of SP in peripheral blood leukocytes of patients with AD by allergens

**Fig. 6:**
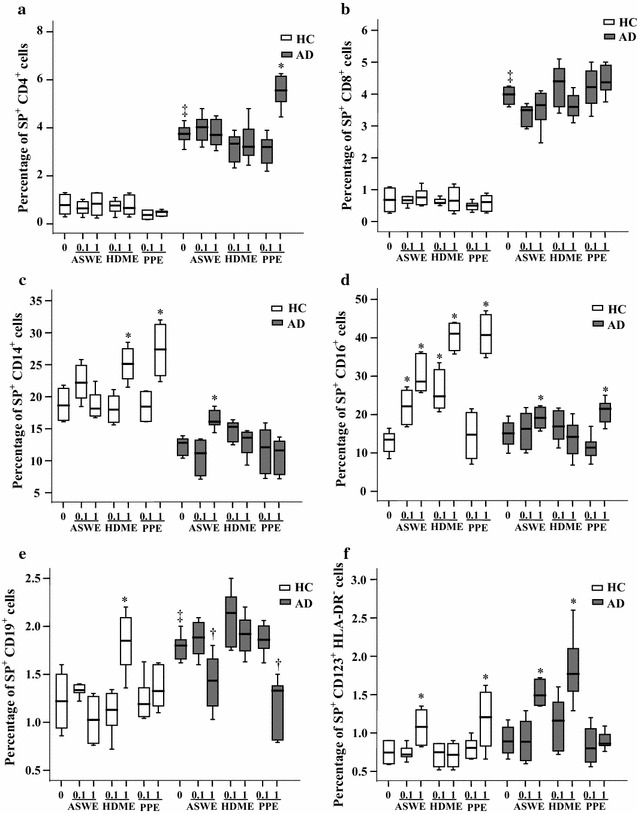
Induction of SP expression in peripheral blood leukocytes by allergens in healthy control (HC) subjects and patients with atopic dermatitis (AD). Cells were challenged by *Artemisia sieversiana* wild allergen extract (ASWE), house dust mite allergen extract (HDME), or *Platanus* pollen allergen extract (PPE) (all at concentrations of 0.1 and 1.0 μg/ml) at 37 °C for 1 h. (*A*), (*B*), (*C*), (*D*), (*E*) and (*F*) represent SP^+^ populations of CD4^+^ (helper T cells), CD8^+^ (cytotoxic T cells), CD14^+^ (monocytes), CD16^+^ (neutrophils), CD19^+^ (B cells), and CD123^+^HLA-DR^−^ cells (basophils) in HC subjects and patients with AD, respectively. The data are displayed as a boxplot for 8 HC subjects or 10 patients with AD, which indicates the median, interquartile range, and the largest and smallest values. *P < 0.05 compared with the increased response to the corresponding medium alone control. ^†^ *P* < 0.05 compared with the decreased response to the corresponding medium alone control. ^‡^ *P* < 0.05 compared with the response to the corresponding medium alone control of HC subject groups (paired Mann–Whitney *U* test)

To confirm if the elevated plasma level of SP in AD may be caused by allergen provocation, we investigated the effects of ASWE, PPE, and HDME allergens on SP expression in peripheral blood leukocytes. The results showed that ASWE enhanced SP expression in CD14^+^, CD16^+^ and CD123^+^HLA-DR^−^ cells of AD patients by 44, 19.6 and 68.5%, respectively, and in CD16^+^ and CD123^+^HLA-DR^−^ cells of HC subjects by 120 and 45%, respectively. HDME exposure increased SP expression only in CD123^+^HLA-DR^−^ cells of AD patients, but it up-regulated SP expression in CD14^+^, CD16^+^ and CD19^+^ cells of HC subjects. Moreover, PPE treatment increased SP expression in CD4^+^ and CD16^+^ cells by 49.3 and 42.4%, respectively, over untreated corresponding cell types in AD patients. PPE incubation also up-regulated the expression of SP in CD14^+^, CD16^+^ and CD123^+^HLA-DR^−^ cells of HC subjects (Fig. 6). In contrast, ASWE and PPE administration clearly down-regulated SP expression in CD19^+^ cells (Fig. 6).

### Induction of expression of NK1R in peripheral blood leukocytes of patients with AD by allergens

**Fig. 7:**
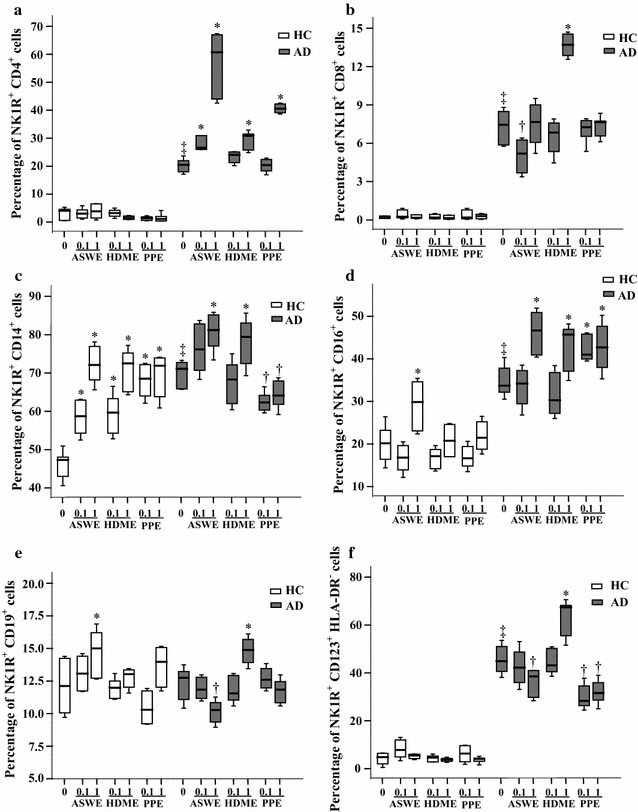
Induction of NK1R expression in peripheral blood leukocytes by allergens in healthy control (HC) subjects and patients with atopic dermatitis (AD). Cells were challenged by *Artemisia sieversiana* wild allergen extract (ASWE), house dust mite allergen extract (HDME), or *Platanus* pollen allergen extract (PPE) (all at concentrations of 0.1 and 1.0 μg/ml) at 37 °C for 1 h. (*A*), (*B*), (*C*), (*D*), (*E*) and (*F*) represent NK1R^+^ populations of CD4^+^ (helper T cells), CD8^+^ (cytotoxic T cells), CD14^+^ (monocytes), CD16^+^ (neutrophils), CD19^+^ (B cells), and CD123^+^HLA-DR^−^ cells (basophils) in HC subjects and patients with AD, respectively. The data are displayed as a boxplot for 8 HC subjects or 10 patients with AD, which indicates the median, interquartile range, and the largest and smallest values. *P < 0.05 compared with the increased response to the corresponding medium alone control. ^†^ *P* < 0.05 compared with the decreased response to the corresponding medium alone control. ^‡^ *P* < 0.05 compared with the response to the corresponding medium alone control of HC subject groups (paired Mann–Whitney *U* test)

To understand the influence of allergens on the expression of NK1R in AD, we investigated the effects of ASWE, PPE, and HDME allergens on NK1R expression in peripheral blood leukocytes. The results showed that ASWE enhanced NK1R expression on CD4^+^, CD14^+^ and CD16^+^ cells of AD patients by 197, 11.7 and 28.2%, respectively, and on CD14^+^, CD16^+^ and CD19^+^ cells of HC subjects by 52.4, 48.1 and 29.9%, respectively. HDME exposure up-regulated NK1R expression in CD4^+^, CD14^+^, CD16^+^, CD19^+^ and CD123^+^HLA-DR^−^ cells of AD patients, but it increased NK1R expression only on CD14^+^ cells of HC subjects. Moreover, PPE treatment increased NK1R expression on CD4^+^ and CD16^+^ cells by 91 and 26.7%, respectively, over untreated corresponding cell types of AD patients. PPE incubation also up-regulated the expression of NK1R on CD14^+^ cells of HC subjects (Fig. 7). However, ASWE administration down-regulated NK1R expression on CD8^+^, CD19^+^ and CD123^+^HLA-DR^−^ cells of AD patients. Similarly, PPE reduced NK1R expression on CD14^+^ and CD123^+^HLA-DR^−^ cells of AD patients (Fig. 7).

### Altered expression of SP in blood leukocytes of OVA-sensitized mice

**Fig. 8:**
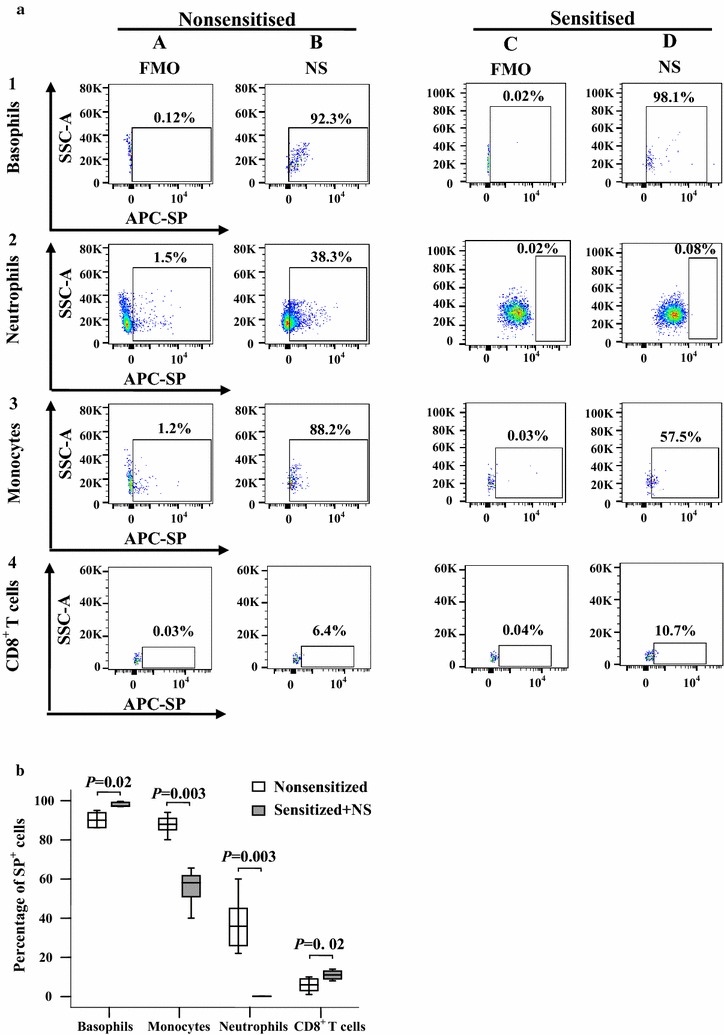
Flow cytometry analysis of expression of substance P (SP) in mouse blood leukocytes. **a** Representative graphs of percentages of SP^+^ leukocytes in the blood of non-sensitized and sensitized mice, respectively. (*A*) and (*C*) are fluorescence minus one (FMO) for SP; (*B*) and (*D*) are all fluorescence (AF) of SP^+^ leukocytes. **b** Demonstrates median percentage values of SP^+^ leukocytes in mouse blood. The data shown are the median (range) value from six different mice. *P < 0.05 in comparison with a corresponding non-sensitized group (paired Mann–Whitney *U* test)

**Fig. 9:**
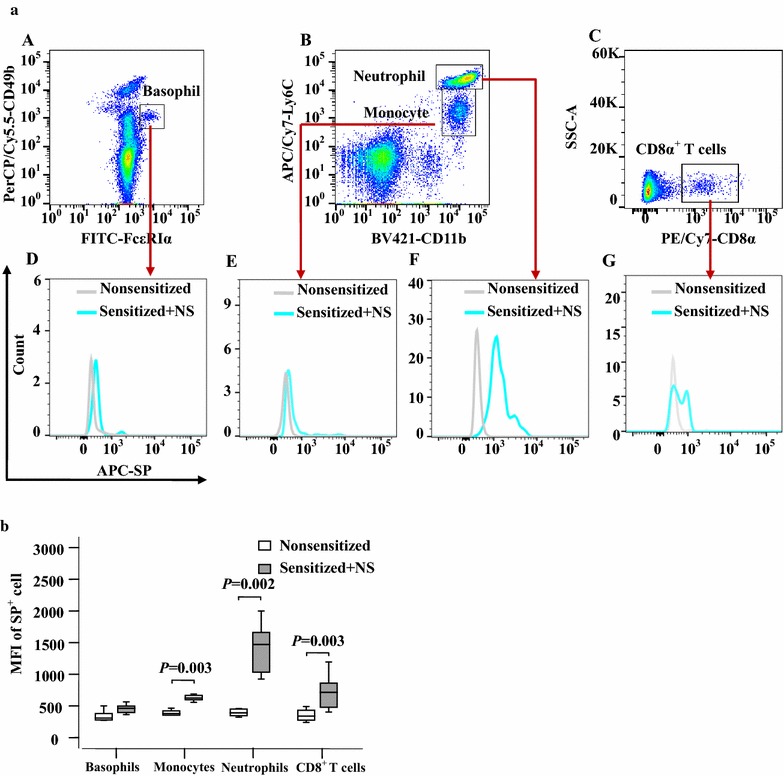
Flow cytometry analysis of changes in mean fluorescence intensity (MFI) of substance P (SP) in mouse blood leukocytes. **a** Representative graphs of the changes in mean fluorescence intensity (MFI) of SP in (*A*) basophils (CD49b^+^FcεRIα^+^), (*B*) monocytes (Ly6C high^+^CD11b^+^) and neutrophils (Ly6C dim^+^CD11b^+^), and (*C*) CD8^+^ T cells. (*D*), (*E*), (*F*), while (*G*) represents SP^+^ blood basophils, monocytes, neutrophils and CD8^+^ T cells in nonsensitized and sensitized mice, respectively. **b** The median values of MFI of SP^+^ cells from six different mice. *P* < 0.05 was considered statistically significant (paired Mann–Whitney *U* test)

To further understand the expression of SP in blood leukocytes under allergic conditions, we investigated the expression of SP in blood leukocytes of OVA-sensitized AD mice by using flow cytometry analysis. The results showed that the proportions of SP-expressing CD8^+^ T cells and basophils were enhanced in the blood of OVA-sensitized mice compared with non-sensitized mice. However, OVA-sensitized mice had smaller SP-expressing monocyte and neutrophil populations than non-sensitized mice (Fig. 8a, b). With regard to the MFI of SP-expressing leukocytes, CD8^+^ T cells, monocytes and neutrophils showed increased SP expression levels in OVA-sensitized mice compared with non-sensitized mice (Fig. 9a, b).

### Altered expression of NK1R in blood leukocytes of OVA-sensitized mice upon SP challenge

**Fig. 10:**
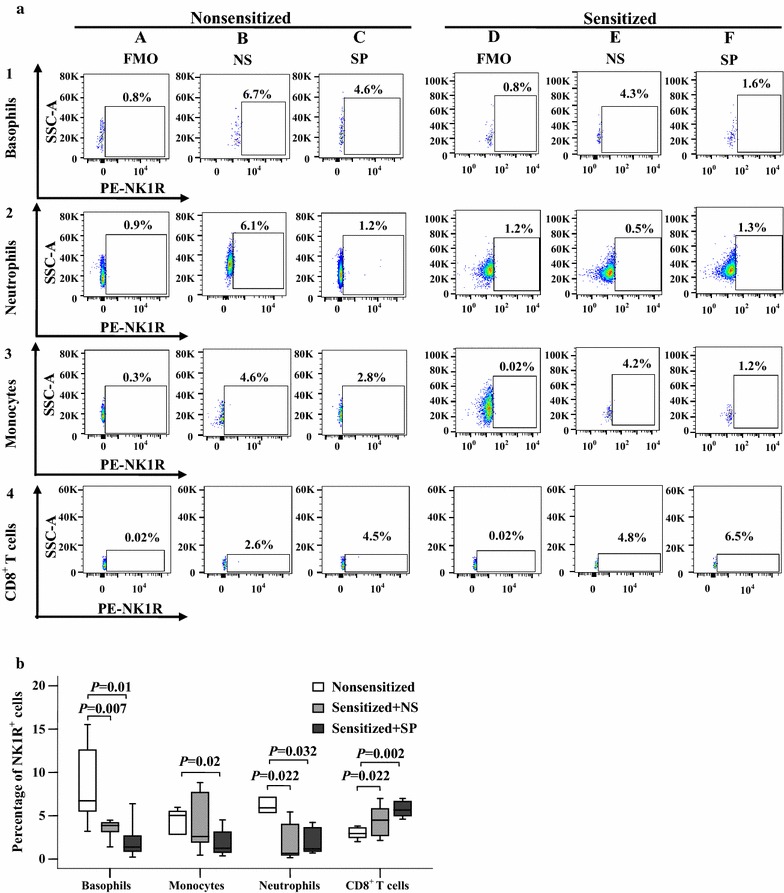
Flow cytometry analysis of expression of NK1R in mouse blood leukocytes. **a** Representative graphs of percentages of NK1R^+^ leukocytes in the blood of non-sensitized and sensitized mice, respectively. (*A*) and (*D*) are fluorescence minus one (FMO) for NK1R; (*B*) and (*E*) are all fluorescence (AF) of NK1R^+^ leukocytes treated with normal saline (NS); (*C*) and (*F*) are all fluorescence (AF) of NK1R^+^ leukocytes treated with SP. **b** Demonstrates median percentage values of NK1R^+^ leukocytes in mouse blood. The data shown are the median (range) value from six different mice. *P < 0.05 in comparison with a corresponding non-sensitized group (paired Mann–Whitney *U* test)

**Fig. 11:**
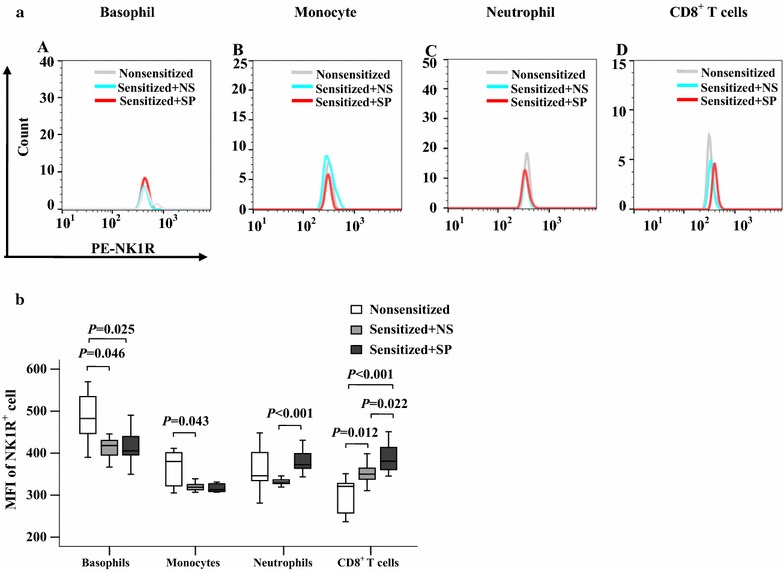
Flow cytometry analysis of changes in mean fluorescence intensity (MFI) of NK1R in mouse blood leukocytes. **a** representative graphs of the changes in mean fluorescence intensity (MFI) of NK1R in (*A*) basophils (CD49b^+^FcεRIα^+^), (*B*) monocytes (Ly6C high^+^CD11b^+^), (*C*) neutrophils (Ly6C dim^+^CD11b^+^), and (*D*) CD8^+^ T cells in nonsensitized mice, sensitized mice treated with normal saline (NS) and sensitized mice treated with substance P (SP), respectively. **b** The median values of MFI of NK1R^+^ cells from six different mice. *P* < 0.05 was considered statistically significant (paired Mann–Whitney *U* test)

To further understand the expression of NK1R in blood leukocytes under allergic conditions and the influence of SP on NK1R expression, we examined the expression of NK1R in blood leukocytes of AD mice in the absence or presence of SP. The results showed that OVA sensitization increased NK1R expressing CD8^+^ T cell populations but decreased NK1R expressing basophil and neutrophil populations. Injection of SP into mouse skin caused an increase in NK1R-expressing CD8^+^ T cell populations but reduced NK1R expressing basophil, monocyte and neutrophil populations (Fig. 10a, b). OVA sensitization also increased the MFI of NK1R on CD8^+^ T cells but decreased the MFI of NK1R on basophils and monocytes. Moreover, injection of SP into mouse skin enhanced the MFI of NK1R expression on CD8^+^ T cells and neutrophils but had little effect on the MFI of NK1R expression on basophils and monocytes (Fig. 11a, b).

## Discussion

For the first time, the current study found up-regulated expression of SP in CD8^+^ T cells of AD, which confirms that not only SP but also CD8^+^ T cells are likely to be involved in the pathogenesis of AD. Since SP is a proinflammatory mediator and CD8^+^ T cells are cytotoxic T cells that secrete an array of proinflammatory cytokines or mediators, including TNF-α, IFN-γ, IL-2 [28, 29], our observation implies that CD8^+^ T cells may contribute to AD via releasing SP.

Past studies of blood T cell phenotyping in patients with AD have provided controversial results [30]. Although Th2-driven inflammation may play a significant role in the pathogenesis of AD [1], a positive correlation between IL-13- and IL-22-producing CD4^+^ and CD8^+^ T cells appears to be a novel finding of AD [30]. Nevertheless, the finding that cutaneous lymphocyte-associated antigen was expressed by a higher percentage of CD8^+^ than CD4^+^ T cells in intrinsic AD cultures seems to further emphasize the importance of CD8^+^ T cells in intrinsic AD [31]. Based on the current observation that up-regulated expression of SP in CD8^+^ T cells of AD, we believe that CD8^+^ T cells are more likely a causative cell type in the development of AD. A study that demonstrates a proinflammatory effect of SP on exacerbation of inflammation via long-term and indirect action on CD8^+^ T lymphocytes may support our hypothesis [32].

As much as a 4.9-fold higher plasma SP level in patients with AD compared to HC subjects strongly implicates that SP is likely to contribute to the pathogenesis of AD. The observation that plasma SP levels in patients with food allergy and drug allergy are not elevated suggests that the enhanced SP level in AD is specific. Previous studies showed that SP plasma levels in AD patients during exacerbation and remission were significantly higher compared to the control group [26] and that patients with AD have a significant increase in the plasma level of SP compared with controls [33], which agree with our current observations.

The increased proportions of SP-expressing CD4^+^, CD16^+^, and CD19^+^ leukocytes in the blood of AD patients is an interesting finding, but the fact that the MFI of CD4^+^, CD16^+^, and CD19^+^ leukocyte expression in the blood of AD patients was lower than that in HC blood remains unclear. These bi-directional results make it difficult to draw a clear conclusion. Therefore, we report that increased expression of SP in CD8^+^ T cells of AD, as both a proportion and an MFI of this cell type, was enhanced in AD blood.

We found enhanced expression of NK1R in CD14^+^ monocytes of AD. For the expression of SP in CD8^+^ T cells of AD, both the proportion and MFI of NK1R expressing CD14^+^ monocytes were enhanced in AD blood. Since proportions of NK1R-expressing CD4^+^, CD8^+^, and CD123^+^HLA-DR^−^ leukocytes in the blood of AD patients are increased, while the MFI of these cell types is not, it is difficult to evaluate their role in AD. Thus, we anticipate that the increased plasma SP may mainly affect blood CD14^+^ cells in patients with AD.

It was reported that upon stimulation with *Dermatophagoides farinae* (Der f), peripheral blood mononuclear cells from AD patients proliferated in a CD80- and CD86-dependent manner, while SP promoted the Der f-induced proliferation in patients with AD [34]. Mite allergens result in localized allergic dermatitis characterized by pronounced epidermal hyperplasia and spongiosis, which was associated with infiltration of eosinophils, neutrophils, degranulated mast cells, CD4^+^ and CD8^+^ T cells, and dendritic cells [17]. However, the influence of allergens on SP and NK1R expression in the blood of AD patients remains uninvestigated. Therefore, for the first time, we investigated the effects of allergens on SP and NK1R expression in the blood of AD patients in the present study. Unexpectedly, ASWE, HDME and PPE failed to up-regulate SP expression in CD8^+^ T cells. Instead, ASWE enhanced SP expression in CD14^+^, CD16^+^ and CD123^+^HLA-DR^−^ leukocytes; HDME exposure increased SP expression only in CD123^+^HLA-DR^−^ cells; and PPE treatment enhanced SP expression in CD4^+^ and CD16^+^ cells of AD patients. These observations suggest that different allergens may affect SP expression in blood leukocytes differently in AD. Another unclear finding was that allergen extracts ASWE, HDME and PPE also elicited up-regulation of expression of SP in some blood leukocytes of HC subjects. Obviously, it is rather hard for us to explain these observations at this stage as **c**ell phase and cell status remain an unsolved issue and experimental challenge in the peripheral blood of human subjects. However, at present, it is not possible to drive these cells into the same phase and same status without affecting their functions.

In contrast, allergens ASWE and HDME both enhanced NK1R expression on CD14^+^ blood leukocytes regardless of whether subjects had AD or HC, and PPE elicited up-regulation of expression of NK1R on CD14^+^ leukocytes of HC subjects, suggesting that allergens are likely to contribute to the development of AD via up-regulating NK1R expression on blood CD14^+^ leukocytes. However, it is difficult to exclude the involvement of other cell types such as basophils in the development of AD as they also express more SP and NK1R upon allergen challenge [13].

OVA-sensitized AD mice showed elevated proportions of SP-expressing CD8^+^ T cells and an increased MFI of SP-expressing CD8^+^ T cells in blood, which agrees with the SP expression situation in human AD blood. However, OVA sensitization had little effect on the proportion of NK1R expression on CD14^+^ cells but decreased the MFI of NK1R expression on monocytes of AD mouse blood, which disagrees with the NK1R expression situation in human AD blood. The explanation for the discrimination between NK1R expression situations on blood monocytes of AD patients and mouse AD model could be that mouse blood monocytes do not respond to allergen OVA challenge [35], while human blood monocytes respond well to allergen challenge [36]. Injection of SP into mouse skin induced an increase in the NK1R-expressing CD8^+^ T cell population and MFI of NK1R expression on CD8^+^ T cells, but it decreased NK1R-expressing basophil, monocyte and neutrophil populations. This is an unexpected result as we expected to see SP up-regulation of NK1R expression on monocytes, as observed in human AD monocytes. Nevertheless, down-regulation of NK1R expression by SP has been previously reported [37], which may support our current observation.

## Conclusions

An elevated plasma SP level, up-regulated expression of SP in CD8^+^ T cells and NK1R expression on monocytes, and allergen-induced up-regulation of expression of SP and NK1R in leukocytes of AD indicate that the SP/NK1R complex is likely to be important in the development of AD. Therefore, SP and NK1R antagonist or blocker agents may help treat patients with AD.

## Abbreviations

AD: atopic dermatitis
MFI: mean fluorescence intensity
NK1R: neurokinin-1 receptor
SP: substance P
ELISA: enzyme-linked immunosorbent assay
HC: healthy control
ASWE: *Artemisia sieversiana* wild allergen extract
HDME: house dust mite allergen extract
PPE: *Platanus* pollen allergen extract
Der f: *Dermatophagoides farina*
